# Rhodamine-Tagged Polymethacrylate Dyes as Alternative Tools for Analysis of Plant Cells

**DOI:** 10.3390/ma15217720

**Published:** 2022-11-02

**Authors:** Rafał Bielas, Justyna Wróbel-Marek, Ewa U. Kurczyńska, Dorota Neugebauer

**Affiliations:** 1 Department of Physical Chemistry and Technology of Polymers, Faculty of Chemistry, Silesian University of Technology, Strzody 9, 44-100 Gliwice, Poland; 2 Institute of Biology, Biotechnology and Environmental Protection, Faculty of Natural Sciences, University of Silesia in Katowice, Jagiellońska 28, 40-032 Katowice, Poland

**Keywords:** rhodamine, fluorescence, cellular localization, Arabidopsis thaliana

## Abstract

A rhodamine B (RhB)-based initiator for atom transfer radical polymerization (ATRP) was synthesized and applied for preparation of poly(2-trimethylammoniumethyl methacrylate) (PChMA), poly(2-hydroxyethyl methacrylate) (PHEMA) and poly(2-trimethylsilyloxyethyl methacrylate) (PHEMATMS). Polymer fluorescence was confirmed by determination of quantum yield by comparative method with piroxicam as the standard exhibiting dependency of emission intensity on the polymer chain hydrophilicity and the kind of solvent. The RhB functionalized polymers were used for biological tests in plant materials except for RhB-PHEMATMS because of weak fluorescence. These two polymers slightly differed in cellular localization. RhB-PChMA was mostly observed in cell walls of root tissues and cotyledon epidermis. It was also observed in cytoplasm and cell organelles of root cap cells and rhizodermis, in contrast with cytoplasm of cotyledon epidermis. RhB-PHEMA was also present in apoplast. A strong signal in protoxylem cell walls and a weak signal in cell walls of rhizodermis and cortex were visible. Moreover, it was also present in cell walls of cotyledon epidermis. However, RhB-PHEMA was mostly observed in cytoplasm and cell organelles of all root tissues and epidermis of cotyledons. Both RhB-polymers did not cause cell death which means that they can be used in living plant material.

## 1. Introduction

Fluorescently labeled polymers bring much attention due to the possibility of employing their miscellaneous physicochemical properties in combination with ability of optical emission [1]. They are less prone to sequestration from cells and tissues as well as usually exhibiting lower toxicity and better performance than low-molecular-weight dyes. [2] A variety of dyes have been used to modify polymers in order to introduce fluorescent properties, such as Nile blue attached to pH-sensing binary polymer brush “corrals” for studies of proton transport in membranes [3] or pH biosensors able to simultaneous monitoring of cells in both near infrared and far red light [4]. The labeling of fluorescein as pendant group in macromolecules of various topologies has been widely reported in the literature [5,6,7]. In other cases, the polymers have been directly prepared from monomers containing fluorescent moieties, for example, anthracene and carbazole [8]. Pyranine has been employed as a polymerization initiator to obtain fluorescent polymethacrylates, which can be used for analyzing apoplastic transport in plants. [9] It has also been applied to modify poly(*L*-lysine) [10] and chitosan [11] for the design of drug delivery systems and intracellular pH sensors.

The other group of common dyes are rhodamines (Rh), which contain a xanthene ring in their structure. They are commonly applied in biology as staining agents, especially in fluorescence microscopy and flow cytometry. Many rhodamine derivatives can be bought under commercial brands, such as Alexa Fluor^®^ or DyLight^®^. Similarly to other dyes, they have also found applications in polymer chemistry for the construction of sensors [12,13] and electroconducting materials [14]. Rh-labeled polyacrylamide has been used to modify ITO electrodes for Au^3+^ detection [15], whereas poly(ether sulfone) nanofibers doped with Rh have indicated high sensitivity and selectivity for Cu^2+^ [16]. The biologically active poly(2-(methacryloyloxy)ethyl phosphorylcholine) has been synthesized with Rh6G-based initiator of atom transfer radical polymerization (ATRP) [17]. There are also reported biological studies on RhB conjugated with poly(ε-caprolactone) to determine both in vivo and in vitro quantitative dynamics of polymer nanoparticles in various animal cells and tissues [18], or oligonucleotides to monitor their uptake by cells [19]. The advantageous high-level microscopic visualization has been supported by RhB labels in poly(vinyl alcohol) hollow microparticles by confocal laser scanning microscopy (CLSM) [20], or poly(ethylene glycol)-*b*-polyglutamate-*b*-polylysine for cholesterol uptake by polymeric vesicles inside living cells via fluorescence lifetime imaging microscopy-Förster resonance energy transfer (FLIM-FRET) method [21]. Additionally, the monomers with RhB pendant group allowed for obtaining fluorescent poly(methyl methacrylate) latex [22]. The properties of polymethacrylate fluorescent markers might be tailor-made to obtain a proper hydrophilicity level or the well-defined molecular weight [23] in contrast to dextrans often used for plant studies, [24] and they do not cause cell cythorrysis [9] unlike popular poly(ethylene glycol)s [25].

In this study, we designed a series of the well-defined polymethacrylates functionalized with RhB, which, after modification to bromoester derivative, was introduced as a new fluorescent initiator. The amphiphilic polymers with different hydrophilicity due to a variety of substituents (N^+^(CH_3_)_3_ vs. OH vs. OSi(CH_3_)_3_) were characterized by spectroscopic methods to confirm their structures, and spectrofluorometric analysis to detect fluorescence, whereas DLS and surface tension measurements were used to evaluate their behavior in aqueous solution. Their marker properties were verified by biological studies on selected plant cells.

Plant protoplasts are enclosed by cell walls. Thus, a cell wall is the first structure of a plant organism that has contact with the environment [26]. Plant cell walls are built of different kinds of polymers such as polysaccharides, structural proteins and in some cases, lignin as well [27]. One of the polysaccharides is cellulose, which forms microfibrils. These microfibrils are embedded in a gel-like matrix composed of non-cellulosic polysaccharides (e.g., hemicelluloses, pectins) [26,27]. Thus, plant cell walls might be compared to a sieve with pores of a definite diameter [28]. Small molecules will be able to diffuse through the pores, but the larger ones will not be able to do so [28]. Moreover, diffusion in cell walls depends not only on the size, but also on the charge of the molecule. Pectins, another main polysaccharide in the walls, are negatively charged and thus can bind cations [29]. That means that positively charged molecules are no longer available for cells. That is why pectins are used as biosorbents for removing toxic metals from waste water [30].

Beneath the cell wall lies the plasma membrane which surrounds cytoplasm with cell organelles. It is a specific structure that plays a vital role in cell growth and development. It is highly selective and its integrity is a key factor that ensures proper cell functioning [31]. In plants, transport through the plasma membrane can occur on three different pathways: (1) diffusion, (2) active transport using carriers, (3) endocytosis and exocytosis [32,33,34,35]. Plasma membrane composition and electric potential play a vital role in transportation of molecules from one side of the membrane to the other side [35,36,37]. A chemical compound that can be regulated not only in size, but also in charge seems an attractive tool not only in the cell wall, but also in plasma membrane analysis. It is advantageous to compare movement of similar-size molecules but with different charge. On the other hand, comparing molecules with different sizes but the same charge is also interesting. In such studies, it is possible to determine whether charge or size plays a more important role in crossing the cell wall and plasma membrane. RhB-polymers can be modified to give us this kind of opportunity. In this study, we focused on determining how charge influences the transport through the cell wall and plasma membrane. Thus, polymers with similar size but different charge (positive and neutral) were used in our studies. The aim of the studies was to check these fluorescent polymers for application in the plant cell study, especially their usefulness for analyzing movement across the plasma membrane and into the cell cytoplasm.

## 2. Materials and Methods

### 2.1. Chemicals

First, 2-(Trimethylsililoxy)ethyl methacrylate and 2-hydroxyethyl methacrylate (HEMATMS and HEMA, Aldrich) were dried under molecular sieves. Second, 2-(*N,N,N*-Trimethylammonium)ethyl methacrylate chloride (ChMA, Aldrich, St. Louis, MI, USA) as an 80% solution in water was dried under reduced pressure until a constant weight was achieved. Rhodamine B chloride (RhB, POCH S.A.), bipyridine (bpy, Sigma-Aldrich) and α-bromoisobutyryl bromide (Sigma-Aldrich, St. Louis, MI, USA) were used without purification. Copper(I) bromide (Sigma-Aldrich) was purified as described in [38]. Methanol, tetrahydrofuran (THF), dimethylformamide (DMF) and other solvents were purchased from Chempur and stored under molecular sieves.

### 2.2. Physicochemical Characterization

The ^1^H NMR spectra of the polymer solutions in DMSO-d_6_ were collected on a Varian Inova 300 MHz spectrometer at 25 °C using TMS as an internal standard. FT-IR spectra were obtained on a Perkin Elmer Spectrum Two spectrometer for solid samples. Dispersity indices (Ð) were determined by size-exclusion chromatography (SEC, Thermo Scientific, Ultimate 3000, Waltham, MA, USA) equipped with an ISO-3100SD isocratic pump, autosampler, degasser, thermostatic box for columns, and differential refractometer RefractoMax 521 Detector. Chromoleon 7.2 Chromatography Data System (CDS, Thermo Scientific) was used for data collection and processing. Pre-column guard 5 µm (50 × 7.5 mm) and two columns: PLGel 5 µm MIXED-C (300 × 7.5 mm) and Malvern Viscotek T6000M (300 × 8 mm) were used for separation. The SEC calibration was based on linear polystyrene standards. The measurements were made in DMF (HPLC grade) as the eluent containing 10 mM LiBr, at 40 °C, with a flow rate of 1 mL/min. Dynamic light scattering (DLS) was performed on a Malvern Zetasizer Nano-S90 and Nano-ZS instruments equipped with an 4mW He−Ne ion laser operating at λ = 633 nm and λ = 532 nm, respectively. Samples of polymer solutions in deionized water (c = 0.055 mg/mL) were placed in PMMA cells, which were next deposited in the thermostated cell compartment of the instrument at 25 °C ± 0.1 °C. All of the sample measurements were performed at fixed scattering angles of 90° and 175°. At least 5 correlation functions were analyzed per sample to obtain an average value. The measurements of surface tension of the polymer solutions were performed using the pendant drop method on the OCA 15EC goniometer (DataPhysics Instruments) with SCA 22 software module (DataPhysics Instruments) for the concentration range 0.0014–0.75 mg/mL. The fluorescence was measured at room temperature in a 10 mm quartz cell using fluoroSENS Pro-11 spectrofluorimeter (Camlin). The polymer solutions (0.055 mg/mL) were prepared in deionized water, DMSO and methanol. The emission spectra were obtained by excitation of polymer samples at the maximum absorption wavelength for RhB at λ_ex_ = 550 nm. The fluorescence quantum yields were calculated using the comparative method with pyroxicam in hexane as a reference (*Φ_ref_* = 0.035) [39] as follows:

$$\Phi_{polymer}=\Phi_{reference}\times \frac{Int_{polymer}}{Int_{reference}}\times \frac{1-10^{-A_{reference}}}{1-10^{-A_{polymer}}}\times \frac{n_{polymer}{}^{2}}{n_{reference}{}^{2}}$$

where *Φ* is the quantum yield, *Int* is the area under the emission peak (on a wavelength scale), *A* is absorbance at the excitation wavelength, and *n* is the refractive index of the solvent [40].

### 2.3. Preparation of RhB Based ATRP Initiator

RhB (1 g; 2.8 mmol) was dissolved in 100 mL of THF and subsequently ethylene glycol (0.32 mL; 5.6 mmol), 4-dimethylaminopyridine (11 mg; 0.09 mmol) and *N*,*N′*-dicyclohexylcarbodiimide (0.62 g; 3 mmol) were added. The mixture was stirred for 24 h. Next, the product was dried under reduced pressure and used without further purification (yield 0.70 g, 64%). The obtained 2-hydroxyethyl derivative of RhB (0.44 g; 0.9 mmol) was dissolved in 20 mL of dry DMF and cooled in an ice bath. Next, α-bromoisobutyryl bromide (136 μL; 1.1 mmol) and triethylamine (154 μL; 1.1 mmol) were added and stirred. After 10 min, the mixture was heated to 100 °C and stirred for 4 h. Next, it was cooled to room temperature and concentrated under vacuum. Dark purple-red solid was purified by column chromatography (stationary phase: silica gel, eluent: chloroform/methanol 3:1). The product-containing fraction was dried under vacuum resulting in metallic purple-red solid (yield 0.41 g, 72%). ^1^H NMR (DMSO-d_6_, δ, ppm): 8.19–8.36, 7.79–7.96, 7.44–7.54, 7.04–7.19, 6.87–7.03 (10H, aromatic rings), 3.58–3.73 (8H, -C**H_2_**-N-), 3.39 (4H, -COO-C**H_2_**-), 1.86 (6H, -C(C**H_3_**)_2_Br, 1.21 (12H, -N(CH_2_-C**H_3_**)_2_).

### 2.4. General Method for RhB-Labelled Polymer Synthesis

The monomer (5 mmol), methanol (1 mL), THF (1 mL), bpy (15.6 mg, 0.1 mmol), and RhB initiator (0.05 mmol) were placed into a Schlenk flask and degassed by two freeze−pump−thaw cycles. The initial sample was taken, and CuBr (7.2 mg, 0.048 mmol) was introduced to the mixture. Next, the reaction flask was immersed into an oil bath at 40 °C. The reaction was stopped after 24 h by exposure to air. Then, the polymer was precipitated in ethyl acetate twice to remove the catalyst. **RhB-PChMA** ^1^H NMR (DMSO-d_6_, δ, ppm): 4.53 (2H, -COO-C**H**_2_-), 3.78 (2H, -C**H_2_**-N^+^-), 3.34 (9H, -N^+^-(C**H_3_**)_3_), 2.03–1.61 (2H, -C**H_2_**- backbone), 1.26–0.61 (3H, -C**H_3_** backbone). **RhB-PHEMA** ^1^H NMR (DMSO-d_6_, δ, ppm): 4.81 (1H, -CH_2_-O**H**), 3.89 (2H, -C**H_2_**-OH), 3.57 (2H, -COO-C**H**_2_), 2.03–1.63 (2H, -C**H_2_**- backbone), 1.08–0.59 (3H, -C**H_3_** backbone). **RhB-PHEMATMS** ^1^H NMR (DMSO-d_6_, δ, ppm): 3.88 (2H, -C**H_2_**-O-), 3.56 (2H, -COO-C**H**_2_), 2.16–1.40 (2H, -C**H_2_**- backbone), 1.1–0.50 (3H, -C**H_3_** backbone), 0.1 (9H, -Si-(C**H_3_**)_3_).

### 2.5. Plant Material

Seedlings of *Arabidopsis thaliana* (ecotype Col-0) were prepared as described before [9]. Briefly, seeds were surface-sterilized for 8–10 min, rinsed three times in water and sown on Petri dishes containing solid 1/2 MS [41] medium with sucrose and agar (pH 5.8). Cultures were kept at 21 °C under a 16 h photoperiod with a light intensity of 40 μmol m^−2^ s^−1^ for 5–8 days.

### 2.6. Fluorochrome and Staining Procedure

Seedlings were placed in aqueous solution (0.05 mg/mL) of each polymer and incubated for 15 min at room temperature. Then, they were rinsed and incubated in 0,01% calcofluor white solution [9]. After that, they were rinsed three times with deionized water and the seedling roots and cotyledons were observed in a confocal laser scanning microscope (CLSM) according to method described earlier [42] or roots were treated with 1M sucrose solution [9] and observed in CLSM. In total, no less than 10 roots and cotyledons from each treatment were analyzed, and the experiments were repeated three times.

### 2.7. Microscopical Observation

At the beginning of the experiments, autofluorescence images of the plant material were collected. For further observations, such microscope settings were selected (laser power, photomultiplier voltage [42]), at which the autofluorescence was not visible in the final image. RhB-PChMA, RhB-PHEMA and RhB-PHEMATMS were excited with a 543 nm wavelength and the emission of these compounds was collected in the range of 585–620 nm. Fluorescein was excited with a 488 nm wavelength and the emission of this compound was collected in the range of 500–530 nm. Calcofluor white was excited with a 405 nm wavelength and the emission was collected in the range of 425–475 nm. The roots and cotyledons were scanned in the Z-axis and individual optical sections were recorded and analyzed in Fluoview (FV10-ASW 4.2 Viewer) and Image J (Fiji) programs.

### 2.8. Cell Viability Detection

To study the influence of fluorescent polymers on cell viability, two tests were performed. The first one employed Evans blue staining [43] and the second one—the fluorescein diacetate (FDA) treatment [44].

## 3. Results and Discussion

### 3.1. Synthesis and Polymer Characterization

**Figure 1:**
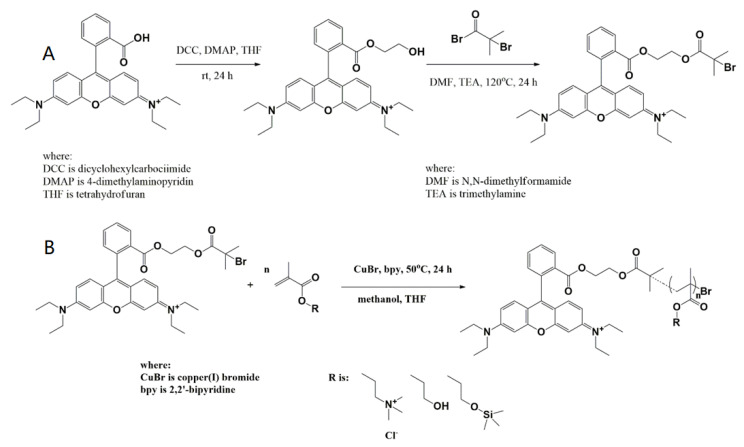
Synthesis of RhB fluorescent initiator (**A**) and its use in ATRP of methacrylates (**B**).

The bromoester derivative of RhB was synthesized in two steps through the introduction of a hydroxyl group and esterification to form the fluorescent initiator (Figure 1A). Both structures were confirmed by ^1^H-NMR spectroscopy with appearing proton signals of −CH_2_-O-CO- at 3.39 ppm and −C(CH_3_)_2_Br at 1.86 ppm, respectively (Figure S1, Supporting information). The final product was able to initiate atom transfer radical polymerization (ATRP) of methacrylates with different levels of hydrophilicity, that is, 2-trimethylammoniumethyl methacrylate chloride (ChMA), 2-hydroxyethyl methacrylate (HEMA) and 2-trimethylsilyloxyethyl methacrylate (HEMATMS), using CuBr/bpy catalyst system and solvent mixture methanol/THF at 50 °C (Figure 1B). The resulting polymers were characterized by relatively low degrees of polymerization (DP = 17–35, Table 1), which were set by initial ratio of monomer to initiator of 100:1, with the intention to obtain small particles of short chains as they are convenient for plant studies. Monomer conversions for DP calculation were determined from ^1^H NMR spectroscopy, which also confirmed structures of polymers (Figure S2, Supporting information) showing signals of protons for characteristic units in each type of polymers, e.g., -CH_2_-N^+^- and -N^+^(CH_3_)_3_ at 4.33 and 3.14 ppm (RhB-PChMA), -CH_2_-OH at 4.81 and 3.89 ppm (RhB-PHEMA) or -Si(CH_3_)_3_ at 0.91 ppm (RhB-PHEMATMS). Additionally, the presence of the functional groups was detected by FT-IR spectroscopy (Figure S3, supporting information).

The ability of amphiphilic polymers to self-assemble was evaluated by determination of critical micelle concentration (CMC). However, the fluorescent labels in polymers excluded the use of fluorescence spectroscopy, which is the standard method to characterize the micellization of polymer systems [45]. For this reason, the surface tension measurements were carried out to estimate CMC values from the plot of logC (where C is concentration in mg/mL) as the cross-over point at low polymer concentration (Figure S4). For more polar RhB-PChMA and RhB-PHEMA, CMC values were determined at 0.085 mg/mL, whereas RhB-PHEMATMS exhibited a double lower value (Table 2).

The behavior of polymers in aqueous solution was also investigated by DLS, which indicated their micellization/aggregation with the formation of relatively big particles. It is likely that the light applied in DLS experiments did excite Rh moieties, resulting in fluorescence. Thus, the scattered light was amplified with the light emitted by particles showing bigger hydrodynamic diameters than the polymeric system actually possesses. This means that the hydrodynamic diameters (d_h_) obtained at light wavelength of 633 nm (66–190 nm) and 532 nm (140–250 nm) were apparent values (Table 2). The lowest values were exhibited for the most hydrophobic RhB-PHEMATMS, which was in contradiction with the CMC results. Additionally, the measurement for RhB-HEMATMS polymer was performed at the concentration above its CMC, that is for aggregated system, thus, the polymer chain might cover fluorescent moieties and prevent the amplification of light scattered. The inconvenience of RhB effect in DLS measurements led us to assume that the real sizes of PChMA and PHEMA particles are lower than the ones determined and they are lower than for PHEMA-TMS. It is probable that the sizes of these particles are similar to sizes of previously studied nanoparticles of polymers functionalized with pyranine [9].

**Figure 2:**
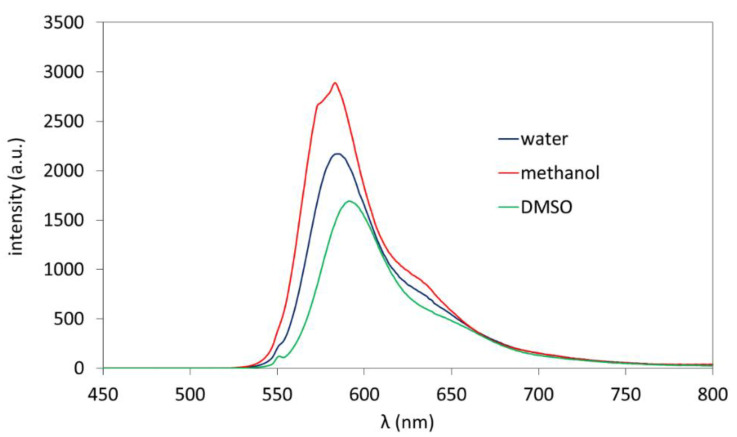
Emission spectra of RhB-PChMA in various solvents.

**Figure 3:**
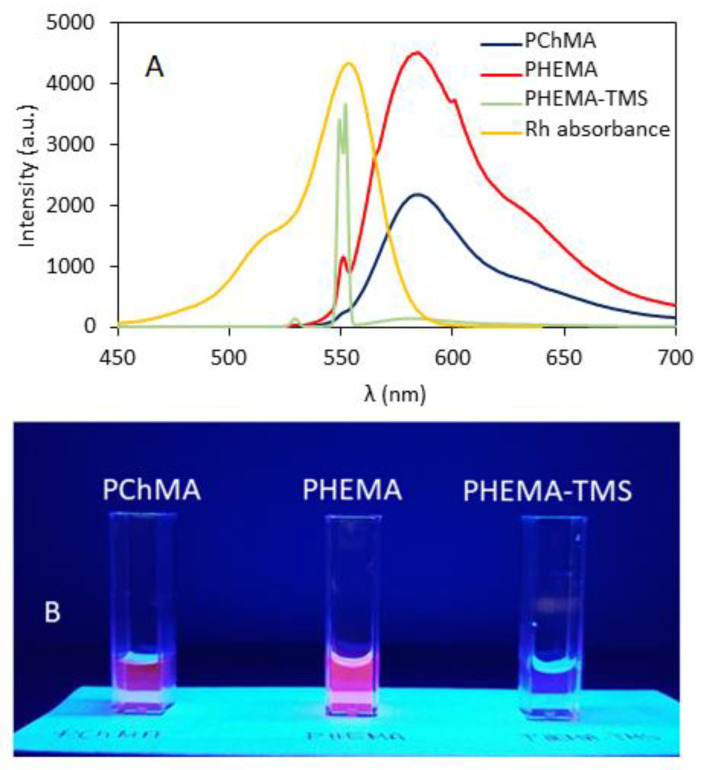
Normalized UV absorbance spectrum of rhodamine B and emission spectra of RhB-labeled polymers in aqueous solution (0.055 mg/mL) (**A**). Fluorescent glowing polymer solutions illuminated with UV lamp at 365 nm (**B**).

Fluorescent properties of the obtained polymers containing RhB starting moieties, which were introduced by novel fluorescent initiator, were investigated by spectrofluorometric measurements. Fluorescence spectra were collected at excitation wavelength λ_ex_ = 550 nm for two groups of samples, i.e., (i) solutions of RhB-PChMA in various solvents (Figure 2) and (ii) solutions of three types of polymers in water (Figure 3). The influence of solvent type on emission intensity of RhB-PChMA indicated that the highest maximum emission wavelength at 540–760 nm (orange-red luminescence) was observed for the sample solubilized in methanol. The emitted fluorescence was reduced in water and DMSO. It is worth mentioning that in the case of previously studied pyranine labeled polymethacrylates [9], the fluorescence emission occurred only in water and methanol, and no emission was observed for DMSO solubilized polymers. This difference may be caused by specific solvent–dye interactions.

Considering the potential application of the polymeric markers in cell biology, water was chosen as the medium for quantum yield analysis. Quantum yields Φ were calculated using comparative method with piroxicam as a fluorescent standard (Table 1). Comparing the emission spectra for various polymers in water (Figure 3), the highest intensity was detected for RhB-PHEMA, whereas almost no emission was observed for RhB-PHEMATMS. The latter polymer with trimethylsilyl groups, as the most hydrophobic in the proposed series, was able to form particles due to probable interactions between TMS and RhB moieties, thus, the emission might be quenched by covering the dye. The most hydrophilic RhB-PChMA with ionic nature exhibited the fastest water solubility, but its emission intensity was twice lower than that of hydroxyl functionalized RhB-PHEMA.

### 3.2. Biological Tests

For biological tests only two polymers were used: RhB-PChMA and RhB-PHEMA. The third polymer (RhB-PHEMATMS) had too weak fluorescence to analyze it in a plant material without causing some damage (by too high laser power needed to visualize it) in plant cells [9].

Two types of plant organs were used in the study: a root and a cotyledon of *Arabidopsis thaliana* seedlings. Analysis concerning the root was performed on two zones: (1) meristematic zone and (2) differentiation zone [46]. These two zones were chosen because of different cell types. In the meristematic zone, cells divide frequently and form a pool of cells which will elongate and differentiate in the following zones. The differentiation zone is built of specialized cells that follow specified developmental programs [46]. In the meristematic zone, the root meristem is surrounded by the root cap. The root cap is built of differentiated cells whose role is to protect the root meristem from injuries during root growth [46]. The root meristem is a group of cells that divide and provide new cells for building the root body [46]. In cotyledons, only epidermis was analyzed, because the fluorescence of RhB-polymers was not visible in mesophyll cells. Cotyledon epidermis is built of two types of cells: (1) pavement cells and (2) guard cells [47,48,49]. In *Arabidopsis thaliana,* pavement cells form a layer of cells that protects inner tissues from environmental factors. They have a puzzle-like shape [50,51]. Guard cells are very specialized, they are responsible for gas exchange and have a kidney shape [49,52]. In plant material, two different localization of fluorescent polymers were observed: apoplastic and cytoplasmic regardless of the plant organ.

**Figure 4:**
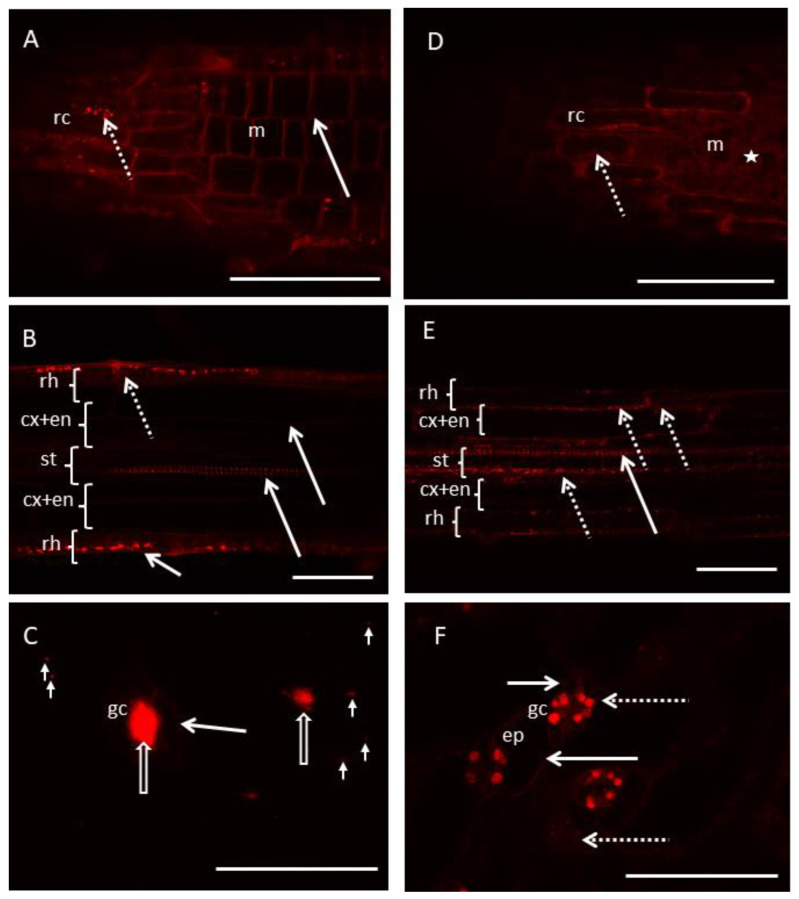
Cellular localization of RhB-polymers in a root (**A**,**B**,**D**,**E**) and cotyledon (**C**,**F**) of *Arabidopsis thaliana*. RhB-PchMA mostly visible in cell walls of root cap and root meristem cells (arrow) and in cell organelles of lateral root cap cells (dotted arrow) (**A**). Apoplastic (arrow) and cellular (dotted arrow) localization of RhB-PchMA observed in the root differentiation zone (**B**). RhB-PchMA present in cell walls of guard cells (arrow) and in the stomatal pore (empty arrow), and as spots on the surface of epidermis (arrowheads) (**C**). RhB-PHEMA visible in the cytoplasm of meristematic cells (asterisk) and root cap cells (dotted arrow) (**D**). In the root differentiation zone, RhB-PHEMA observed in cytoplasm (dotted arrows) of rhizodermis, cortex, endodermis, stele and in cell walls (arrow) of protoxylem (**E**). In a cotyledon epidermis, RhB-PHEMA visible in cytoplasm of pavement cells and in cytoplasm and chloroplasts of guard cells (dotted arrows) (**F**). It was also visible in cell walls of pavement cells and guard cells (arrows) (**F**). Images are Z-stack representations of 30–40 (**A**–**E**) or 2 (**F**) optical sections combined into one picture. Additionally, images A and D are longitudinal tangential sections, so root cap and meristematic cells are simultaneously visible. cx+en-cortex and endodermis, ep-pavement cells of epidermis, gc-guard cell, m-meristematic cells, rc-root cap, rh-rhizodermis, st-stele (pericycle, phloem, xylem and procambium). Scale bar: 50 um.

In the meristematic zone of the root, RhB-PChMA was present only in the cell walls of meristematic cells (Figure 4A, arrow). However, in root cap cells, the polymer was visible in cell walls and cytoplasm (generally in cell organelles; Figure 4A, dotted arrow). Similarly, RhB-PChMA was observed in cell walls of rhizodermis, cortex, endodermis and protoxylem of root differentiation zone (Figure 4B, arrows). However, cytoplasmic localization in rhizodermis was also visible (Figure 4B, dotted arrow). In both root zones, cytoplasmic localization was observed only in the root cap and rhizodermis, while apoplastic localization was observed in each tissue of the root (Table 3). Thus, it can be interpreted that positively charged RhB-PChMA was bound in cell walls by negatively charged pectins. There is also the possibility that plasma membrane permeability might be different in tissues that have direct contact with the environment (root cap and rhizodermis) compared with internal tissues of the root. However, both assumptions require further study.

In the case of cotyledons RhB-PChMA was present in cell walls of guard cells (Figure 4C, arrow) and in stomatal pores (Figure 4C, empty arrows). Moreover, there were also visible spots of that polymer on the surface of pavement cells (Figure 4C, arrowheads). Obtained results can be explained by the interaction of pectins with RhB-PchMA, but confirmation of pectin participation in this process needs further studies. Moreover, presence of cuticle on the cotyledon surface might be an additional barrier for RhB-PchMA penetration into the cotyledon and it might be a cause of polymer adsorption on the cotyledon surface. Presence of RhB-PchMA in the stomatal pore is an unexpected result and needs further investigation.

The *RhB-PHEMA* was present only in cytoplasm of root cap and meristematic cells (Figure 4D, dotted arrow and asterisk, respectively). It was absent in apoplast of meristematic zone tissues (Table 3). Cytoplasmic localization of RhB-PHEMA was also visible in the root differentiation zone where rhizodermis, cortex, endodermis and stele showed presence of this polymer not only in cytoplasm but also in cell organelles (Figure 4E, dotted arrows). Moreover, apoplastic localization of RhB-PHEMA was visible in protoxylem (Figure 4E, arrow). These results show that RhB-PHEMA as a neutral particle can enter cell cytoplasm regardless of the tissue type. Moreover, its lack of apoplast of meristematic zone cells suggests that RhB-PHEMA is not bound by cell walls or that this binding is weak. This is confirmed by very weak fluorescence of RhB-PHEMA in cell walls of the root differentiation zone (Figure S5E). The exception is protoxylem with strong fluorescent signal, which suggests that neutral particles can also be bound by cell wall structures. However, we cannot determine the character of this binding for now.

In cotyledons, RhB-PHEMA was observed in cytoplasm (and organelles) of guard cells and pavement epidermal cells (Figure 4F, dotted arrows). This polymer was also visible in cell walls of both types of epidermal cells (Figure 4F, arrows). Once again, neutral particles penetrates plant material better than particles with positive charge (Table 3). This is probably because more polymer is available for further penetration (as it is not bind by cell wall components or the binding is weak). Thus, more polymer can cross the plasma membrane. Additionally, it is possible that charge on the plasma membrane surface allows neutral particles to enter the cytoplasm, while negatively charged molecules are prevented from entering.

#### 3.2.1. Additional Test

Described localizations of both RhB-polymers were confirmed by staining plant material with calcofluor white. Calcofluor white is an agent which stains cellulose in plant cell walls [53]. We stained plant material with calcofluor white after RhB-polymers treatment. Then, presence of co-staining was checked. However, a close vicinity of the cell wall and the cytoplasm precludes unambiguous determination whether the signal is only in the cell wall or in the cell wall and in the cytoplasm. Thus, besides calcofluor white staining also plasmolysis (using 1 M sucrose solution) was performed. As it is known, a plant cell contracts its cytoplasm in response to plasmolyticum. The cell wall and cytoplasm are visible as two different boundaries which helps to confirm the apoplastic or cytoplasmic localization of the RhB-polymers (Figure S5A,B,D,E).

As it was mentioned before, both RhB-polymers have a similar size and the same fluorochrome but slightly different cellular localization. These differences might result from the cell wall structure. Among components that built plant cell walls, there is pectin, which is known as anionic polysaccharide that binds cations [29,30]. Thus, it is possible that the apoplastic localization of Rhb-PChMA was due to interaction of this polymer with pectin in plant cell walls. Another thing that might influence Rhb-PChMA localization is plasma membrane potential. It is known that charged molecules can cross the plasma membrane only if there are specific channels. Moreover, the direction of movement depends on electrochemical potential of plasma membrane. Maybe Rhb-PChMA remained in cell walls because of plasma membrane potential prevents its movement into cytoplasm [54]. There is also the possibility of crossing the plasma membrane in the process called endocytosis. Relatively large molecules can enter into the cell in vesicles. This could explain how such large molecules such as RhB-polymer can cross plasma membrane. However, there is no explanation for the movement of these polymers within the pores of cell walls if they really have such a large size. This is the reason why we think that the sizes obtained in our studies are apparent. More probable is that that sizes of RhB-polymers are similar to the Pyranine-polymers described and studied earlier [9]. There is also the possibility that RhB-polymer may unfold in plant material. We assume that chains of both polymers fold and create a sphere. However, this is the case in solvent solution. Plant cells are very specific. They have walls packed with charged molecules. The plasma membrane has also its own electrochemical potential. These factors can influence the level of convolutedness of polymers. Thus, the real size of particles can differ from the results obtained in the solvent. That is why this issue requires further study.

#### 3.2.2. Cell Viability Tests

Only living cells were taken into consideration in these studies. To check the viability of cells, we used two different dyes: Evans blue and fluorescein diacetate (FDA). Evans blue is a dye which marks only dead cells. These cells are dark blue in a bright field microscope because the plasma membrane is ruptured [43] and the dye enters the cytoplasm [55]. Fluorescein diacetate is a fluorochrome that has two acetate groups. When FDA enters the cytoplasm, these two groups are cleaved by endogenous esterases and fluorescein is released into cytoplasm. Only fluorescein is fluorescent, so visible green fluorescence indicates that the plant cell is alive because enzymes are active [44]. After RhB-polymer treatment, plant material was incubated in FDA and checked for cell viability. RhB-polymers did not cause cell death (Figure S5C,F). In some cases, the root cap cells were dead (Figure S5C), however, this might be a result of mechanical injury, or programmed cell death (PCD) process that normally occurs in these cells [56].

## 4. Conclusions

Rhodamine B was modified to obtain ATRP initiator, which was successfully used in the synthesis of fluorescent polymethacrylates. Their various hydrophilicity had a major impact on both spectral and nano characteristics. Lower critical micellization concentration (and thus possible aggregation) was detected for the most hydrophobic polymer PHEMATMS. The latter yielded the lowest sizes of particles in DLS of aqueous solution, whereas the d_h_ values for particles of PChMA and PHEMA were apparent as it was indicated by biological studies demonstrating their movement through the walls into the plant cells. The quantum yield and fluorescence intensity decreased with increasing hydrophobicity. The influence of the solvent on the fluorescence intensity showed that methanol is the best solvent for this type of material, but respectable intensity was also provided in water, which is a crucial solvent for biological studies of the designed polymers. Testing RhB-polymers in plant material yielded the following results:

(1) Both RhB-polymers did not cause cell death, thus, they can be used in living plant material;
(2) Different localizations of RhB-polymers depended on the charge. Positively charged RhB-PChMA was present mostly in cell walls, while neutral RhB-PHEMA was observed mostly in cytoplasm. These results suggest the differential interaction of these polymers with living cells;
(3) The molecular mechanisms of such diversity are unknown and need further studies.

## Figures and Tables

**Table 1:** Characteristics of obtained fluorescent polymers.

	DP	M_nNMR_ (g/mol)	Ð	Φ
RhB-PChMA	17	3500	1.01	0.15
RhB-PHEMA	28	3650	1.26	0.18
RhB-PHEMATMS	35	7100	1.75	0.06

Conditions: [Monomer]_0_:[RhBInitiator]_0_:[CuBr]_0_:[bpy]_0_ = 100:1:1:2, methanol:THF = 1:1 vol. ratio, 50 °C, 24 h; DP is polymerization degree calculated by ^1^H NMR; Ð is the dispersity index as the molecular weight distribution determined by SEC; Φ is a quantum yield of fluorescence.

**Table 2:** Characteristics of polymer nanoparticles in solution.

	d_h_ (nm) λ = 633 nm	d_h_ (nm) λ = 532 nm	CMC (mg/mL)
RhB-PChMA	190	210	0.085
RhB-PHEMA	166	250	0.086
RhB-PHEMATMS	66	140	0.04

**Table 3:** Cellular localization of RhB-polymers.

Rhb-PChMA			RhB-PHEMA	
Root	cytoplasm	cell wall	cytoplasm	cell wall
Root cap ^1^	95% (35 ^a^/37 ^b^)	95% (35 ^a^/37 ^b^)	98% (58 ^a^/59 ^b^)	0% (0 ^a^/59 ^b^)
Meristematic cells ^1^	0% (0 ^a^/37 ^b^)	95% (35 ^a^/37 ^b^)	98% (58 ^a^/59 ^b^)	0% (0 ^a^/59 ^b^)
Rhizodermis ^2^	97% (36 ^a^/37 ^b^)	97% (36 ^a^/37 ^b^)	93% (55 ^a^/59 ^b^)	93% (55 ^a^/59 ^b^) *
Cortex and endodermis ^2^	38% (15 ^a^/37 ^b^)	76% (28 ^a^/37 ^b^)	93% (55 ^a^/59 ^b^)	93% (55 ^a^/59 ^b^) *
Stele ^3^	38% (15 ^a^/37 ^b^)	86% (32 ^a^/37 ^b^)	85% (50 ^a^/59 ^b^)	83% (49 ^a^/59 ^b^)
Cotyledon	cytoplasm	cell wall	cytoplasm	cell wall
Pavement cells	0% (0 ^a^/35 ^b^)	91% (32 ^a^/35 ^b^)	86% (30 ^a^/35 ^b^)	86% (30 ^a^/35 ^b^)
Guard cells	0% (0 ^a^/35 ^b^)	91% (32 ^a^/35 ^b^)	86% (30 ^a^/35 ^b^)	86% (30 ^a^/35 ^b^)

^1^—Cells/tissues present in root apical meristem; ^2^—Cells/tissues present in root differentiation zone; ^3^—In the differentiation zone, the stele is built from pericycle, phloem, xylem and procambium cells. ^a^—Number of roots/cotyledons where fluorescent polymers were visible; ^b^—Number of roots/cotyledons that were analyzed. *—Only weak fluorescence was visible.

## Data Availability

Not applicable.

## Supplementary Materials

The following supporting information can be downloaded at: https://www.mdpi.com/article/10.3390/ma15217720/s1, ^1^H-NMR spectra of RhB initiator and its precurors; ^1^H-NMR and FT-IR spectra of polymers: RhB-PChMA, RhB-PHEMA, and RhB-PHEMATMS; CMC of polymers based on surface tension measurements; cellular localization of RhB-polymers and viability of *Arabidopsis thaliana* root cells.
