# Supplementary material for: Rhodamine-Tagged Polymethacrylate Dyes as Alternative Tools for Analysis of Plant Cells
Source: Materials (Basel). 2022 Nov 2;15(21):7720. doi: 10.3390/ma15217720 (PMC9658429; doi:10.3390/ma15217720)
Supplement: Supplementary file 1 [file materials-15-07720-s001.zip › materials-1995371-supplementary.pdf]

Supplementary materials

Rhodamine-Tagged Polymethacrylate Dyes as Alternative Tools for Analysis of Plant Cells

Rafał Bielas [1], Justyna Wróbel-Marek [2,*], Ewa U. Kurczyńska [2] and Dorota Neugebauer [1,*]

[1] Department of Physical Chemistry and Technology of Polymers, Faculty of Chemistry, Silesian University of Technology, Strzody 9, 44-100 Gliwice, Poland
[2] Institute of Biology, Biotechnology and Environmental Protection, Faculty of Natural Sciences, University of Silesia in Katowice, Jagiellońska 28, 40-032 Katowice, Poland
* Correspondence: justyna.wrobel@us.edu.pl (J.W.-M.); dneugebauer@polsl.pl (D.N.)

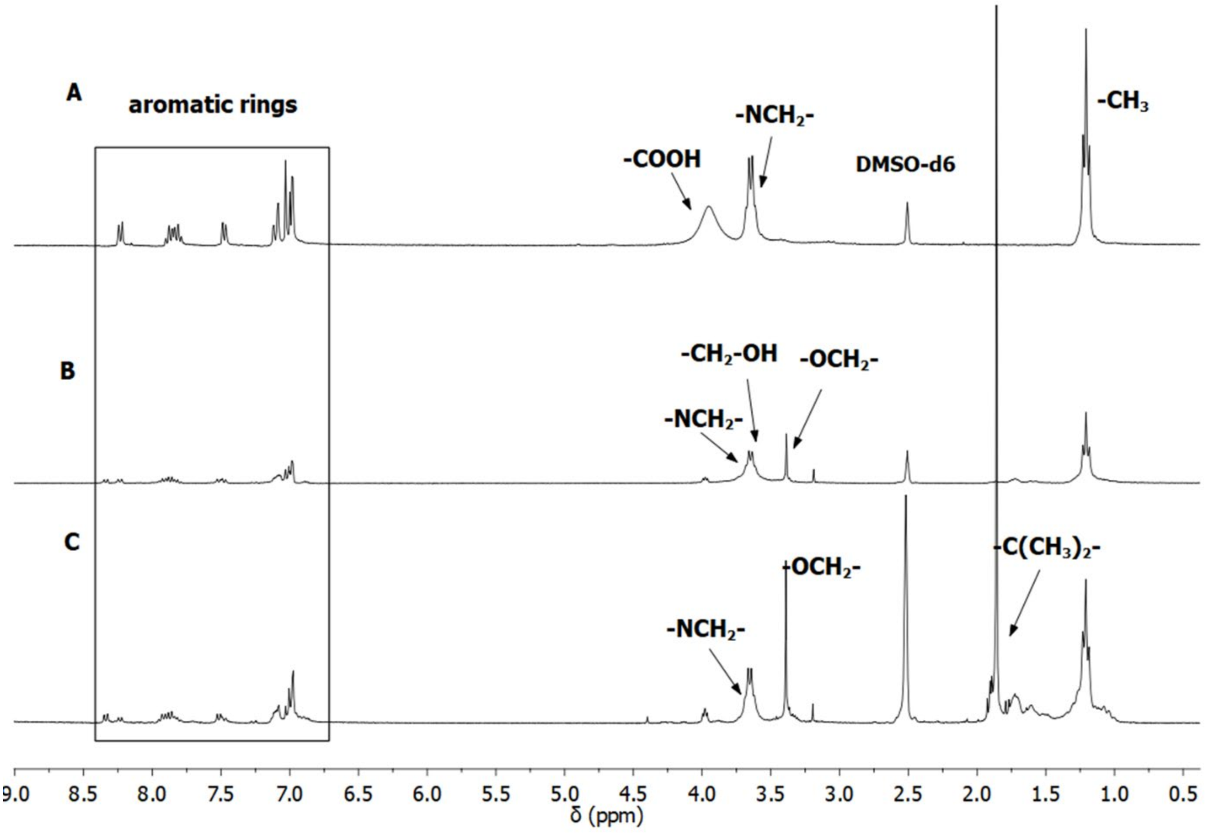

Figure S1. ^{1}H-NMR spectra of RhB (A), its 2-hydroxyethyl derivative (B) and bromoester functionalized initiator (C).

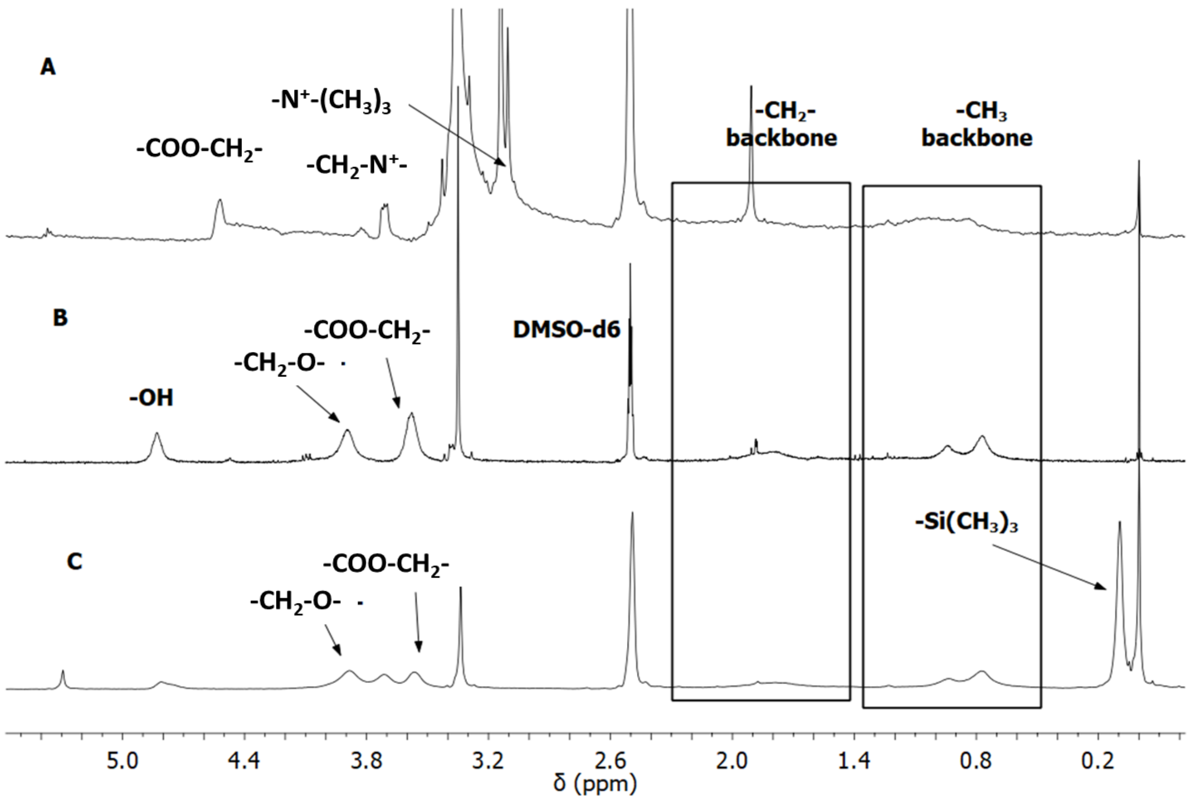

Figure S2. ^{1}H-NMR spectra of polymers: RhB-PChMA (A), RhB-PHEMA (B), and RhB-PHEMATMS (C).

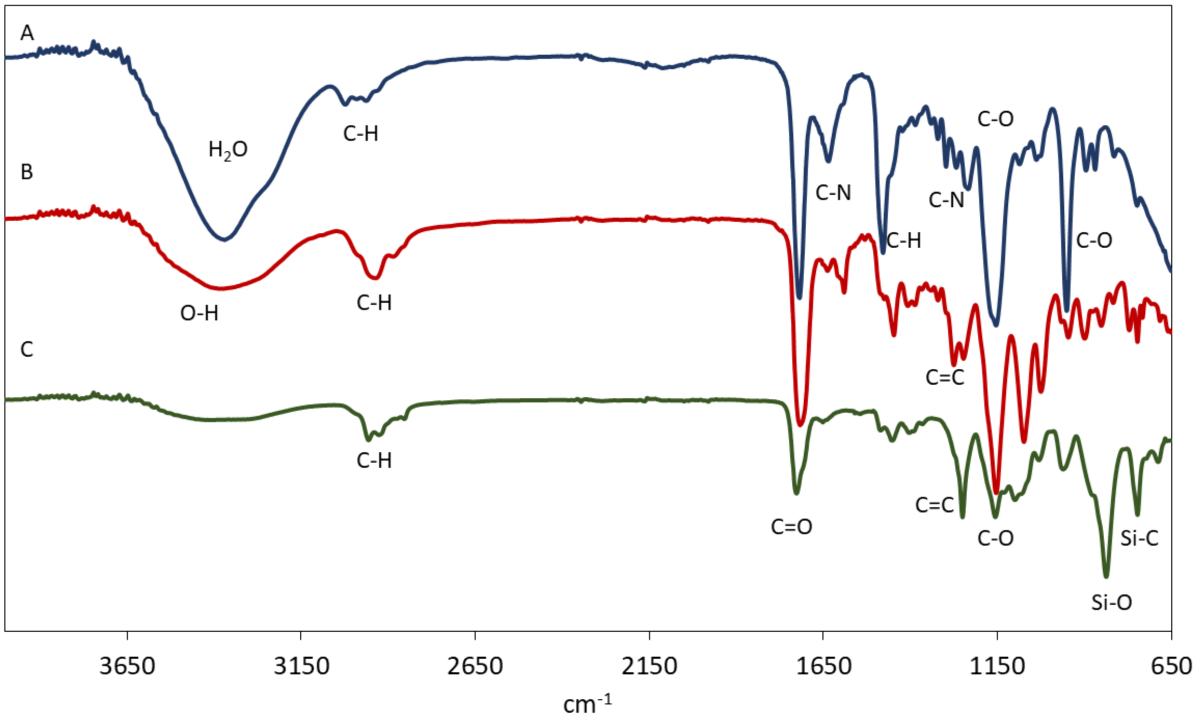

Figure S3. FT-IR spectra of polymers of RhB-PChMA (A), RhB-PHEMA (B), and RhB-PHEMA-TMS (C).

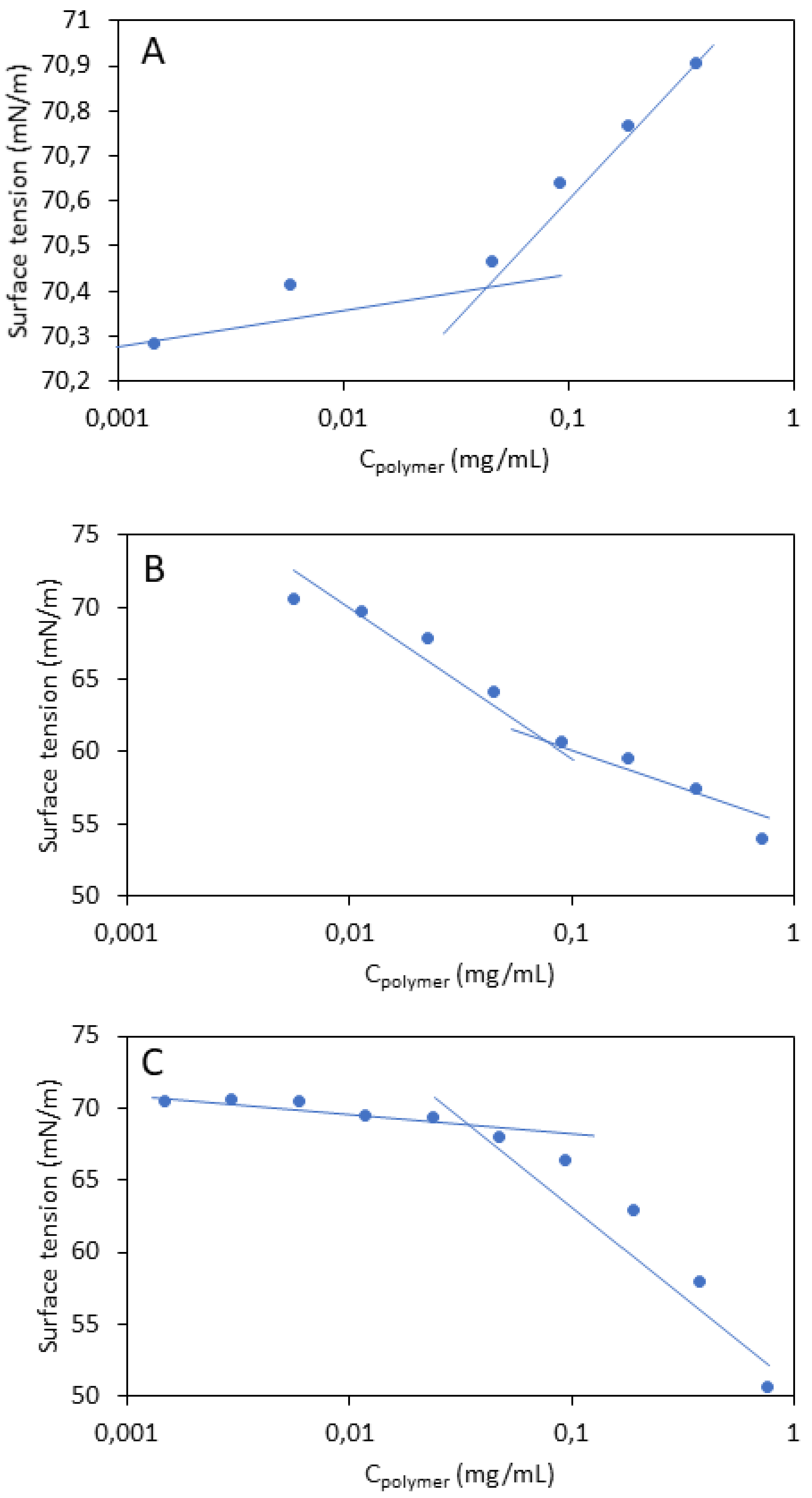

Figure S4. CMC of polymers based on surface tension measurements for RhB-ChMA (A), RhB-HEMA (B), and RhB-HEMATMS (C).

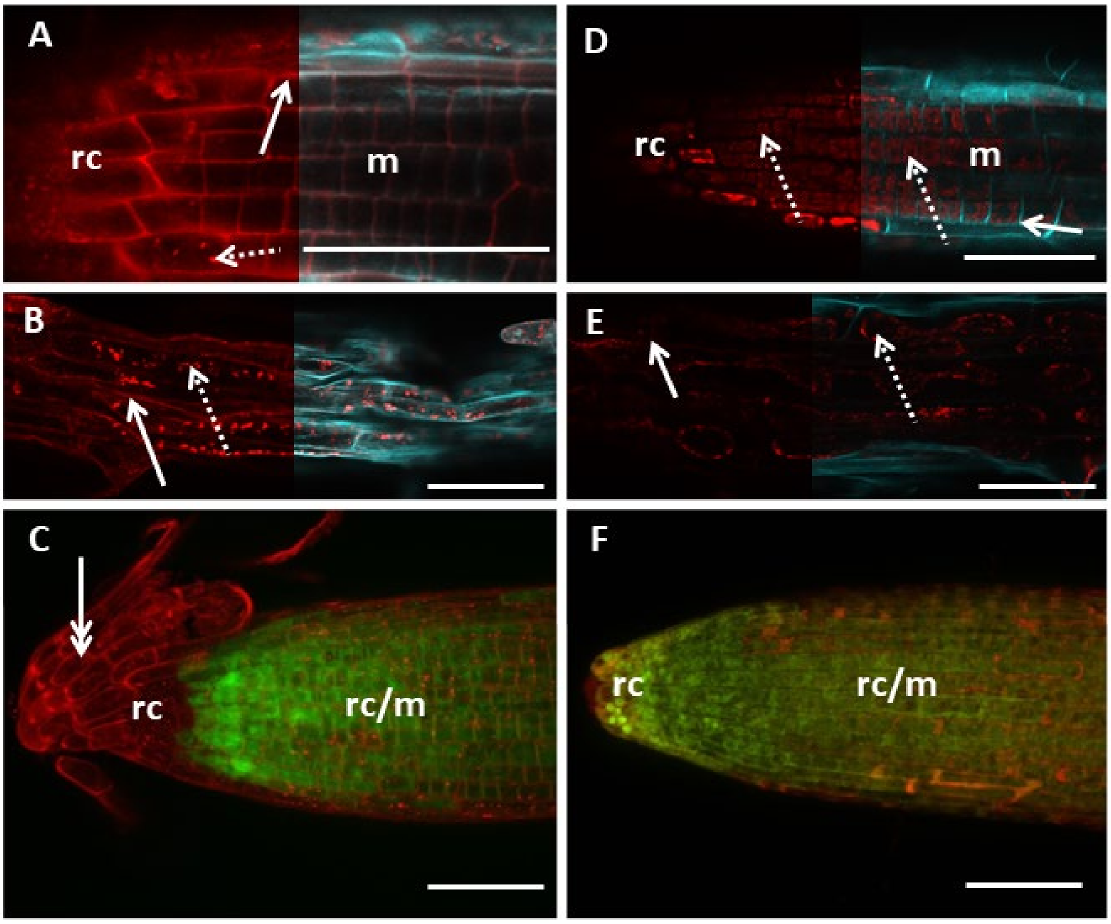

Figure S5. Cellular localization of RhB-polymers and viability of *Arabidopsis thaliana* root cells. Apoplastic localization (arrow) of RhB-PChMA (red) in meristematic zone (which was plasmolysed with 1M sucrose) was confirmed by calcofluor white (blue) which marks celulose in plant cells (red and blue signals overlaps) (A). Double-stained (RhB-PChMA and calcofluor white) roots were also plasmolysed with 1M sucrose to confirm apoplastic localization (arrow) of RhB-PChMA in differentation zone of the root (B). Moreover, cytoplasmic localization (dotted arrow) of RhB-PChMA was also observed, but only in root cap cells and rhizodermis (A, B, respectively). Usage of FDA showed that meristem cells were viable after RhB-PChMA treatment (fluorescein – green, RhB-PChMA – red), however some root cap cells (especially detaching root cap cells; double arrow) were dead (lack of green fluorescence) (C). Cellular localization of RhB-PHEMA (red) in meristematic zone cells (plasmolysed with 1M sucrose) was visible in double-stained roots where calcofluor white (blue) stained cell walls (arrow) and RhB-PHEMA was present only in cytoplasm (dotted arrow) (D). Moreover, after plasmolysis of the root (in 1M sucrose) cytoplasmic localization (dotted arrow) of this polymer was also visible in cells of root differentation zone (E). However, weak apoplastic signal (arrow) was also visible in rhizodermis and cortex cells of root differentiation zone (E). FDA test showed that meristematic and root cap cells were viable after RhB-PHEMA treatment (fluorescein – green, RhB-PHEMA – red) (F). All images are Z-stack representation of 30-40 optical sections combined into one picture. In pictures A, B, D, E left side shows only polymer staining and rigth side shows RhB-polymer and calcofluor white staining. m-meristematic cells, rc/m-root cap cells and meristematic cells. Scale bar: 50µm.
